# Efficacy and safety of ERAS protocols in pediatric laparoscopic appendectomy: a systematic review and meta-analysis

**DOI:** 10.3389/fped.2026.1862189

**Published:** 2026-08-05

**Authors:** Di Wang, Rongkun Zhu, Xiaoxuan Ma, Jing Zhao, Cong Shang, Xiwei Hao

**Affiliations:** Department of Pediatric Surgery, The Affiliated Hospital of Qingdao University, Qingdao, China

**Keywords:** enhanced recovery after surgery, eras, laparoscopic appendectomy, meta-analysis, pediatrics

## Abstract

**Background:**

Acute appendicitis is the most common surgical emergency in children, and laparoscopic appendectomy has become the standard of care. Enhanced recovery after surgery (ERAS) protocols have demonstrated significant benefits in adult populations, but their application in pediatric emergency surgery remains less established.

**Objective:**

To systematically evaluate the efficacy and safety of ERAS protocols compared with traditional perioperative care in children undergoing laparoscopic appendectomy for acute appendicitis.

**Methods:**

A systematic search was conducted in PubMed, Web of Science, Cochrane Library, Embase, and China National Knowledge Infrastructure (CNKI) from inception to March 2026. Randomized controlled trials (RCTs) comparing ERAS protocols with traditional perioperative care in pediatric patients undergoing laparoscopic appendectomy were included. The primary outcomes were postoperative hospital stay and time to first flatus; secondary outcomes included postoperative pain scores and total complications. Meta-analysis was performed using RevMan 5.4, with mean differences (MDs) for continuous outcomes and risk ratios (RRs) for dichotomous outcomes. Heterogeneity was assessed using the I^2^ statistic.

**Results:**

Eight RCTs comprising 1,135 pediatric patients were included. ERAS protocols significantly reduced postoperative hospital stay (MD: −2.04 days, 95% CI: −2.74 to −1.34, *P* < 0.00001, I^2^ = 96%), accelerated time to first flatus (MD: −7.51 h, 95% CI: −11.00 to −4.03, *P* < 0.0001, I^2^ = 99%), alleviated postoperative pain (MD: −1.70 on VAS 0–10, 95% CI: −2.72 to −0.68, *P* = 0.001, I^2^ = 99%), and reduced the risk of total postoperative complications (RR: 0.29, 95% CI: 0.15 to 0.58, *P* = 0.03, I^2^ = 54%). Sensitivity analyses confirmed the robustness of the findings.

**Conclusions:**

ERAS protocols are effective and safe for children undergoing laparoscopic appendectomy, significantly reducing hospital stay, accelerating gastrointestinal recovery, alleviating postoperative pain, and lowering complication risk. These findings support broader adoption of ERAS in pediatric emergency surgical practice, while acknowledging that some components may already be integrated into routine care in certain settings, and that further optimization through day-surgery pathways is warranted for uncomplicated cases.

## Introduction

Acute appendicitis represents the most common surgical emergency in the pediatric population, affecting approximately 70,000 children annually in the United States alone (1, 2). The surgical management of pediatric appendicitis has undergone significant evolution over the past two decades, with laparoscopic appendectomy now established as the standard of care due to its well-documented advantages over open surgery, including reduced postoperative pain, shorter hospital stays, lower wound infection rates, decreased intra-abdominal adhesion formation, and faster return to normal activities (3). The clinical spectrum of pediatric appendicitis ranges from uncomplicated (simple) to complicated forms (gangrenous, perforated, or abscess formation), with the latter associated with substantially higher morbidity and prolonged recovery periods (4).

The Enhanced Recovery After Surgery (ERAS) concept, initially introduced by Kehlet in the 1990s, represents a paradigm shift in perioperative care through the integration of multiple evidence-based interventions aimed at reducing surgical stress, maintaining physiological function, and accelerating postoperative recovery (4). While ERAS protocols have been profoundly impactful in adult surgical populations for nearly three decades, demonstrating consistent reductions in complications, length of stay, and healthcare costs across various surgical specialties (5), their adoption in pediatric surgery has been comparatively slower, beginning approximately 10 years ago (3). The multidisciplinary approach inherent to ERAS—encompassing preoperative optimization, standardized anesthesia protocols, minimally invasive surgical techniques, multimodal analgesia, and early postoperative mobilization and nutrition—has gradually gained momentum in pediatric care (3).

Recent years have witnessed significant advancements in pediatric ERAS, with early single-center studies demonstrating promising reductions in both complications and length of stay for pediatric patients (3, 6). The first ERAS Society recommendations specifically tailored for neonatal perioperative care were published in 2024, marking a critical milestone in standardizing perioperative care for surgical neonates (7). Furthermore, two multicenter trials—the Pediatric Urology Recovery After Surgery Endeavor (PURSUE) and the Enhanced Recovery In Children Undergoing Surgery (ENRICH-US)—have completed enrollment and are expected to publish results in 2025, which will further strengthen the evidence base (3). However, despite these advances, the application of ERAS principles in emergency pediatric surgical conditions, particularly acute appendicitis, remains less established (8). While several key ERAS domains may be implementable in emergency settings given consolidated evidence levels, the peculiarities of pediatric populations and the technical diversity of appendectomy procedures necessitate condition-specific evaluation (8). Pediatric appendicitis presents unique challenges, including the need for urgent intervention and the varying disease severity (8, 9).

Several recent systematic reviews have addressed aspects of ERAS in pediatric surgery (10), and a scoping review published in 2017 identified significant gaps in the literature regarding recovery optimization after laparoscopic appendectomy, calling for more randomized controlled trials on regional anesthesia and perioperative care in children (11). However, to date, no comprehensive meta-analysis has specifically synthesized the available evidence on ERAS protocols in pediatric laparoscopic appendectomy across the full spectrum of disease severity. Therefore, we conducted this systematic review and meta-analysis of randomized controlled trials to evaluate the efficacy and safety of ERAS protocols compared with traditional perioperative care in children undergoing laparoscopic appendectomy for acute appendicitis. We hypothesized that ERAS implementation would significantly reduce postoperative hospital stay, accelerate gastrointestinal function recovery, alleviate postoperative pain, and decrease complication rates without increasing readmission risk.

## Methods

### Protocol and registration

This systematic review and meta-analysis was conducted in accordance with the Preferred Reporting Items for Systematic Reviews and Meta-Analyses (PRISMA) 2020 guidelines (12). The protocol was registered with the International Prospective Register of Systematic Reviews (PROSPERO) under registration number CRD420261355367.

### Search strategy

We systematically searched five major electronic databases: PubMed, Web of Science, Cochrane Library, Embase, and China National Knowledge Infrastructure (CNKI) from inception to March 2026. The search strategy combined keywords and medical subject headings related to three core concepts: (1) “enhanced recovery after surgery” OR “ERAS” OR “fast-track surgery” OR “accelerated rehabilitation”; (2) “appendectomy” OR “appendicectomy” OR “appendicitis”; (3) “child” OR “pediatric” OR “paediatric” OR “children”. No language restrictions were applied. The reference lists of included studies and relevant systematic reviews were manually screened to identify additional eligible studies.

### Inclusion and exclusion criteria

Studies were included if they met the following PICOS criteria:

#### Population

Children (aged ≤18 years) undergoing laparoscopic appendectomy for acute appendicitis (including both uncomplicated and complicated forms).

#### Intervention

Application of ERAS protocols during the perioperative period, encompassing preoperative, intraoperative, and postoperative care elements.

#### Comparison

Traditional perioperative care without ERAS elements.

#### Outcomes

At least one of the following outcomes reported: postoperative hospital stay, time to first flatus/defecation, postoperative pain scores (VAS), and postoperative complications (including wound infection, intra-abdominal abscess, adhesive intestinal obstruction, nausea, and vomiting). Study design: Randomized controlled trials (RCTs).

Exclusion criteria were: (1) non-randomized studies, quasi-randomized trials, cohort studies, case-control studies, case series, reviews, conference abstracts, or letters; (2) studies involving open appendectomy without laparoscopic approach; (3) studies including patients with contraindications to laparoscopy or requiring conversion to open surgery; (4) studies with insufficient data for extraction or meta-analysis.

### Study selection and data extraction

Two reviewers independently screened titles and abstracts of all retrieved records. Full texts of potentially eligible studies were then assessed independently by both reviewers against the inclusion criteria. Any disagreements were resolved through discussion or consultation with a third reviewer. The study selection process was documented in a PRISMA flow diagram.

Data extraction was performed independently by two reviewers using a standardized data extraction form. The following information was extracted from each included study: (1) study characteristics (first author, year of publication, country, sample size, patient demographics); (2) details of ERAS interventions and comparator treatments; (3) outcome data as specified above. For continuous outcomes, means and standard deviations were extracted; if medians and interquartile ranges were reported, they were converted to means and standard deviations using established methods. For dichotomous outcomes, the number of events and total participants in each group were extracted.

### Risk of bias assessment

Two reviewers independently assessed the risk of bias of included RCTs using the Revised Cochrane Risk of Bias tool for randomized trials (RoB 2.0) (13). The RoB 2.0 tool evaluates five domains: (1) bias arising from the randomization process; (2) bias due to deviations from intended interventions; (3) bias due to missing outcome data; (4) bias in measurement of the outcome; (5) bias in selection of the reported result. Each domain was judged as “low risk of bias,” “some concerns,” or “high risk of bias.” An overall risk of bias judgment was derived for each study based on the domain-level assessments. Disagreements between reviewers were resolved through discussion or consultation with a third reviewer. The results of the risk of bias assessment were presented in summary tables and figures.

### Outcome measures

The primary outcomes of this meta-analysis were:

Postoperative hospital stay: Defined as the duration from surgery to discharge (in days or hours).

Time to first flatus/defecation: Defined as the interval from surgery to the first passage of flatus or stool (in hours), reflecting recovery of gastrointestinal function.

Secondary outcomes included:

Postoperative pain scores: Measured using the Visual Analogue Scale (VAS, range 0–10) at various time points (6 h, 12 h, 24 h postoperatively). For meta-analysis, we prioritized 24 h scores when available; otherwise, the time point closest to 24 h was extracted.

Postoperative complications: Including total complications, wound infection, intra-abdominal abscess, adhesive intestinal obstruction, nausea, and vomiting.

### Statistical analysis

All statistical analyses were performed using Review Manager (RevMan) version 5.4 (Cochrane Collaboration, Copenhagen, Denmark). For continuous outcomes, mean differences (MDs) with 95% confidence intervals (CIs) were calculated when outcomes were measured using the same scale (with units specified as days for hospital stay, hours for time to first flatus, and points for VAS pain scores); standardized mean differences (SMDs) with 95% CIs were used when different scales were employed. For dichotomous outcomes, risk ratios (RRs) with 95% CIs were calculated.

Heterogeneity among studies was assessed using the Cochrane Q test (*χ*^2^) and the I^2^ statistic. I^2^ values of 25%, 50%, and 75% were considered to represent low, moderate, and high heterogeneity, respectively. A fixed-effects model was used when heterogeneity was low (I^2^ < 50%); otherwise, a random-effects model was applied.

Sensitivity analyses were conducted by sequentially excluding each study to assess the robustness of the pooled estimates, with particular attention to studies rated as having “some concerns” in the risk of bias assessment.

Publication bias was assessed using funnel plots and Egger's test when at least 10 studies were included in the meta-analysis for a given outcome. A two-sided *P*-value < 0.05 was considered statistically significant.

## Results

### Study selection and characteristics

**Figure 1:**
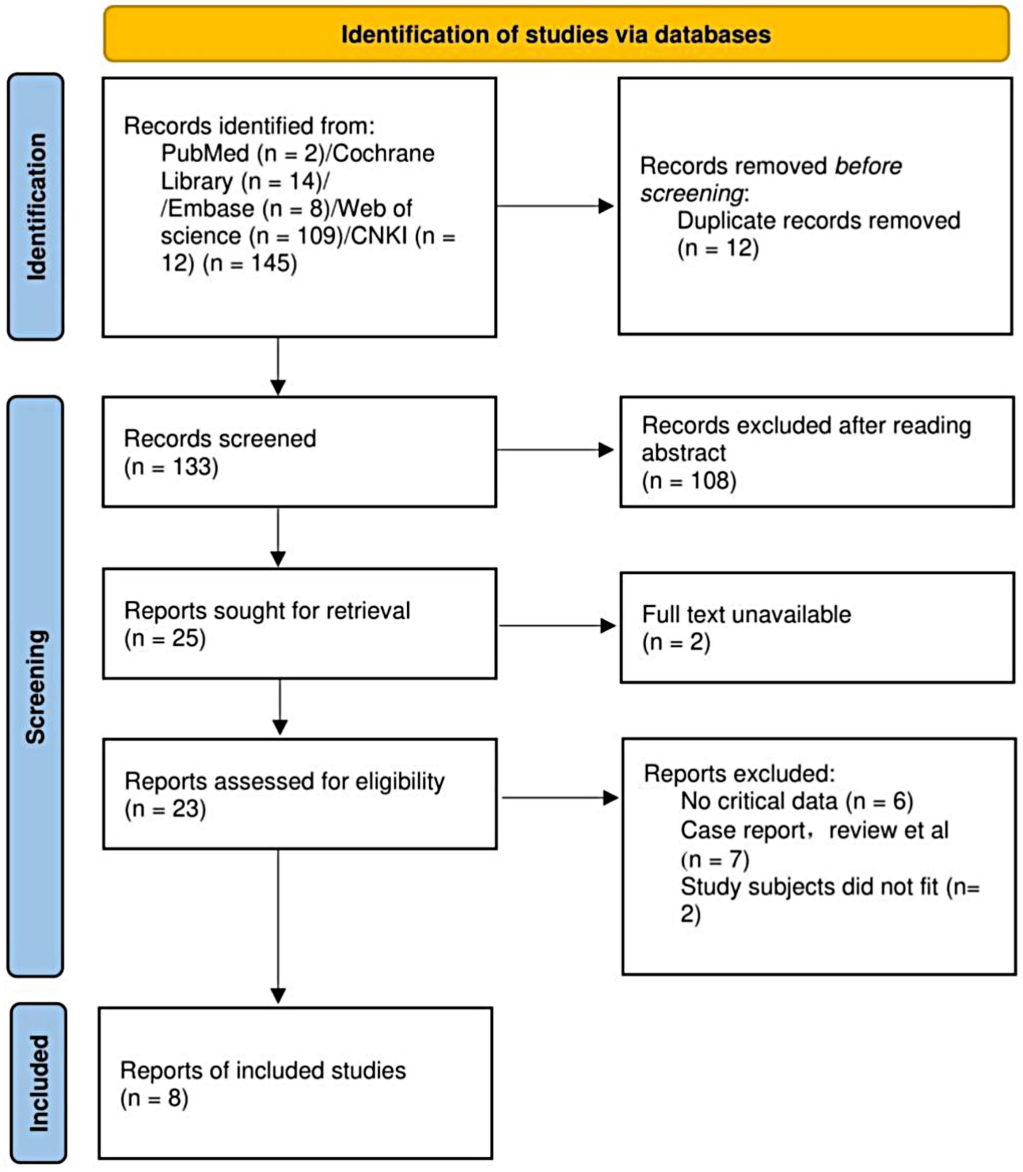
PRISMA flow diagram of study selection process.

A total of 145 potentially relevant records were identified through database searching. After removal of duplicates, 133 records underwent title and abstract screening, of which 108 were excluded as irrelevant to the study question. The remaining 23 full-text articles were assessed for eligibility, and 15 were excluded with reasons. Finally, 8 randomized controlled trials (14–21) were included in this meta-analysis. The PRISMA flow diagram illustrating the study selection process is shown in Figure 1.

The baseline characteristics of the included studies (14–21) are summarized in Table 1. All studies were conducted in China and published between 2019 and 2025. Sample sizes ranged from 82 to 248 participants.

**Table 1:** Baseline characteristics of included studies.

Study	Country/Region	Sample Size (ERAS/Control)	Age Range (years)	Appendicitis Type	ERAS Core Interventions	Control Group Management
Wu (14)	China	124/124	5–14	Complicated (gangrenous, perforated, abscess)	Preoperative carbohydrate loading 2 h before surgery; no routine gastric/urinary catheters; intraoperative warming; multimodal analgesia (ropivacaine local infiltration + lappaconitine patch + ibuprofen); oral intake 6 h postoperatively; early ambulation; traditional Chinese medicine “Chang Zhan Lian Fang”	Traditional fasting and fluid restriction; routine gastric/urinary catheters; no specific warming measures; on-demand analgesia; oral intake after flatus; conventional activity guidance
Zeng (15)	China	60/60	3–14	Uncomplicated (day surgery)	Preoperative glucose drink 2 h before surgery; no gastric/urinary catheters; midazolam anesthesia induction; ropivacaine local infiltration before incision + postoperative transvs. abdominis plane block; intraoperative warming; ambulation and oral intake 6 h postoperatively; liquid diet on postoperative day 1	Fasting for 8 h; routine gastric/urinary catheters; no specific warming; no preemptive analgesia; oral intake after flatus; activity encouraged but not mandatory
Zhao (16)	China	103/105	3–14	Acute (unspecified)	Child Life Specialist intervention (preoperative model play, postoperative “little train” game) added to conventional care; other ERAS measures not detailed	Conventional care (preoperative education, postoperative wound care, dietary guidance, etc.)
Yu (17)	China	42/42	3–12	Mixed (simple, suppurative, gangrenous)	Fasting 4 h, fluid restriction 1 h, oral GNS 2 h preoperatively; short half-life anesthetics; intraoperative warming, fluid restriction; no drainage tube; intradermal suture; non-steroidal analgesia; oral intake 6 h postoperatively with gradual progression; bed mobilization 6 h postoperatively, early ambulation	Traditional fasting and fluid restriction; unrestricted fluid infusion; routine drainage tube; opioid analgesia; oral intake after flatus; activity based on patient preference
Liu (18)	China	77/77	3–10	Acute (unspecified)	Ultrasound-guided quadratus lumborum block (ropivacaine); fasting 4 h, fluid restriction 2 h, preoperative functional drink 30 min before surgery; parental accompaniment during anesthesia induction; laryngeal mask anesthesia; early postoperative ambulation and oral intake	Traditional fasting 6 h, fluid restriction 4 h; tracheal intubation anesthesia; no nerve block; routine postoperative analgesia
Chen (19)	China	43/40	4–14	Uncomplicated	Preoperative family health education, respiratory function training; intraoperative warming; early postoperative oral intake (electrolyte solution), early activity (bed exercises, four-step ambulation protocol); multimodal analgesia (ropivacaine + lappaconitine patch)	Routine care (not detailed)
Zhang SM (20)	China	54/62	6–14	Acute (simple, suppurative)	Preoperative ERAS education; shortened fasting (solids 8 h, milk 6 h, breast milk 4 h, glucose water 2 h preoperatively); no routine gastric tube; intraoperative incisional local anesthesia; warming; oral sugar water 6 h postoperatively with gradual progression; prophylactic oral ibuprofen; ambulation 6 h postoperatively	Traditional fasting and fluid restriction; routine gastric tube; no specific analgesia; oral intake after flatus; activity encouraged
Zhang JG (21)	China	62/60	1–14 (months)	Complicated (suppurative, perforated)	Fasting 6 h, glucose water 2 h preoperatively; avoidance of bowel preparation; parental accompaniment during anesthesia induction; intraoperative warming, no drainage tube; incisional ropivacaine injection; postoperative acetaminophen suppository analgesia; bed activity 6 h postoperatively, ambulation on day 1; oral intake 6 h after awakening	Traditional fasting 12 h, fluid restriction 6 h; enema and gastric tube when necessary; no specific warming; on-demand analgesia; oral intake after flatus; ambulation on day 1

### Risk of bias assessment

**Figure 2:**
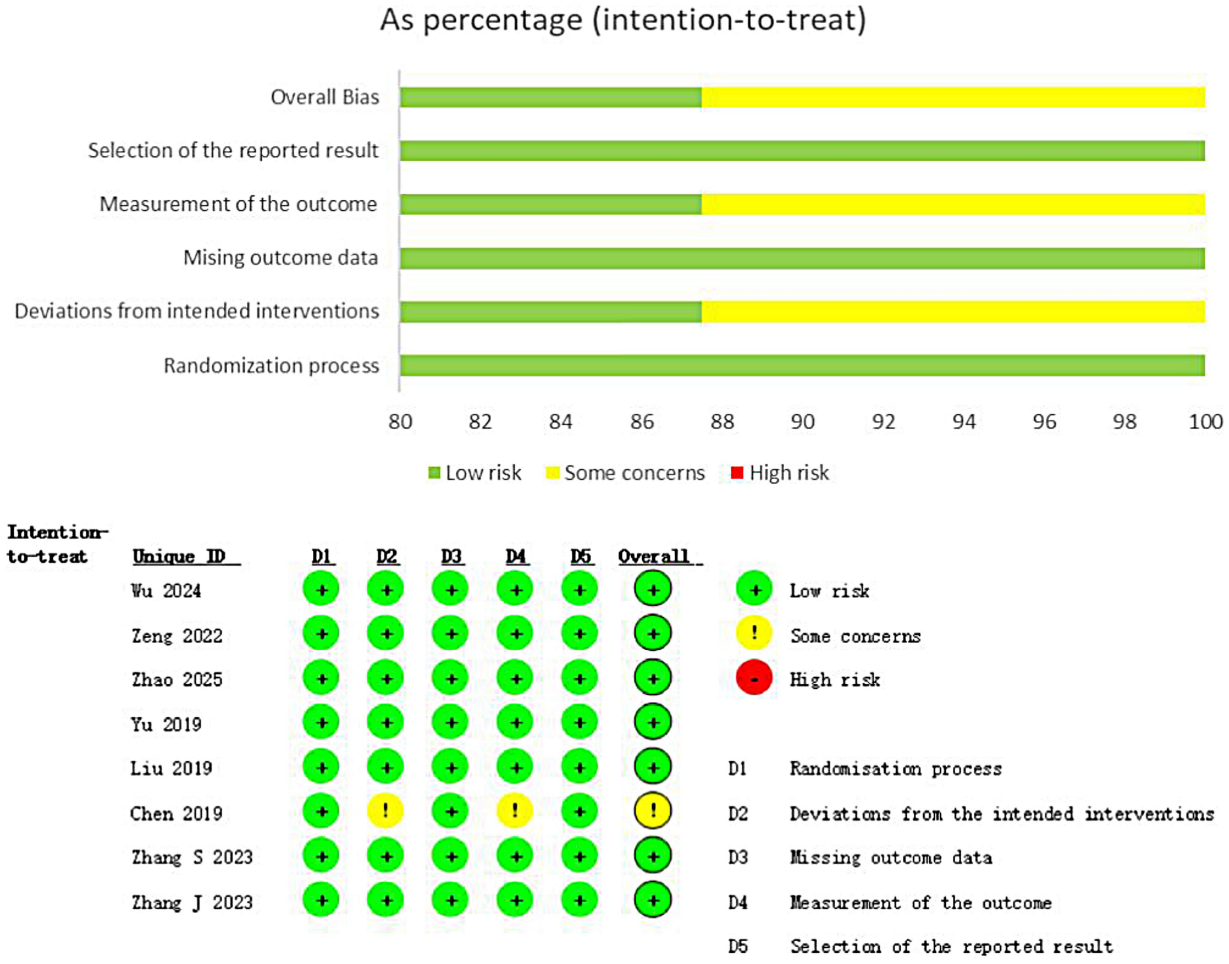
Risk of bias assessment using the RoB 2.0 tool.

The risk of bias assessment using the RoB 2.0 tool is presented in Figure 2. Overall, seven studies (14–18, 20, 21) were judged as having low risk of bias, and one study (19) was judged as having some concerns due to insufficient detail regarding intervention implementation fidelity and the use of unblinded subjective outcome measures.

### Primary outcomes

#### Postoperative hospital stay

**Figure 3:**
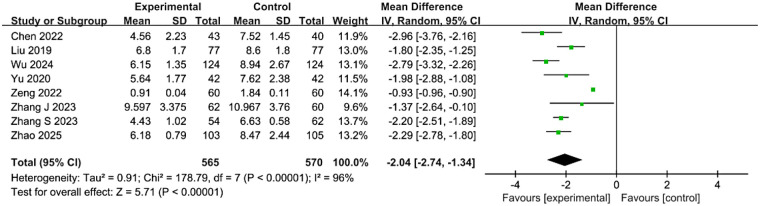
Forest plot for postoperative hospital stay (days).

All eight studies (14–21) reported postoperative hospital stay. The pooled analysis using a random-effects model (due to high heterogeneity) demonstrated that ERAS protocols significantly reduced postoperative hospital stay compared with traditional care, with a mean difference of −2.04 days (95% CI: −2.74 to −1.34, *P* < 0.00001; Figure 3). Substantial heterogeneity was observed among studies (I^2^ = 96%, *P* < 0.00001). Sensitivity analysis by sequential exclusion of each study did not significantly alter the pooled estimate, with MD ranging from −1.82 to −2.21, indicating the robustness of the findings.

### Time to first flatus

**Figure 4:**
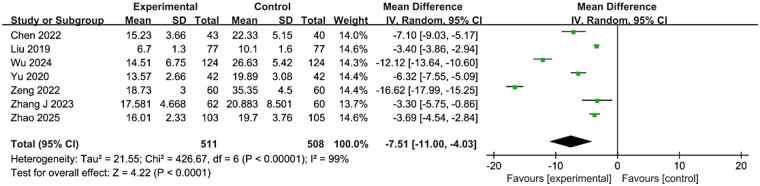
Forest plot for time to first flatus (hours).

Seven studies (14–19, 21) provided data on time to first flatus. One study (20) reported time to first defecation rather than flatus and was excluded from this analysis to maintain clinical consistency. The random-effects meta-analysis showed that ERAS protocols significantly accelerated gastrointestinal function recovery, with a mean difference of −7.51 h (95% CI: −11.00 to −4.03 h, *P* < 0.0001; Figure 4). High heterogeneity was detected (I^2^ = 99%, *P* < 0.00001). Sensitivity analysis confirmed the stability of the results, with pooled MD ranging from −6.89 to −8.12 after sequential exclusion of individual studies.

### Secondary outcomes

#### Postoperative pain scores

**Figure 5:**
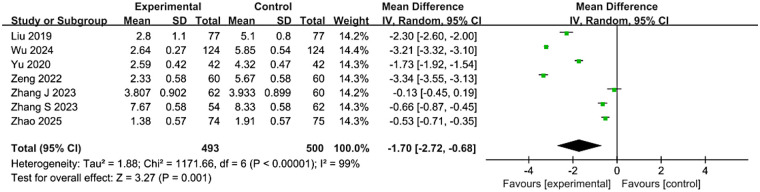
Forest plot for postoperative pain scores (points on VAS 0-10).

Seven studies (14–18, 20, 21) reported postoperative pain scores using the Visual Analogue Scale (VAS, 0–10), with measurement time points ranging from 6 h to 24 h postoperatively across studies. One study (19) did not report pain outcomes. The random-effects meta-analysis demonstrated that ERAS protocols significantly reduced postoperative pain, with a mean difference of −1.70 points (95% CI: −2.72 to −0.68, *P* = 0.001; Figure 5). Significant heterogeneity was observed (I^2^ = 99%, *P* < 0.00001). Sensitivity analysis excluding the study with the highest VAS scores did not substantially change the overall effect (MD: −1.52, 95% CI: −2.48 to −0.56), indicating that the finding was not driven by a single outlier study.

#### Postoperative complications

**Figure 6:**
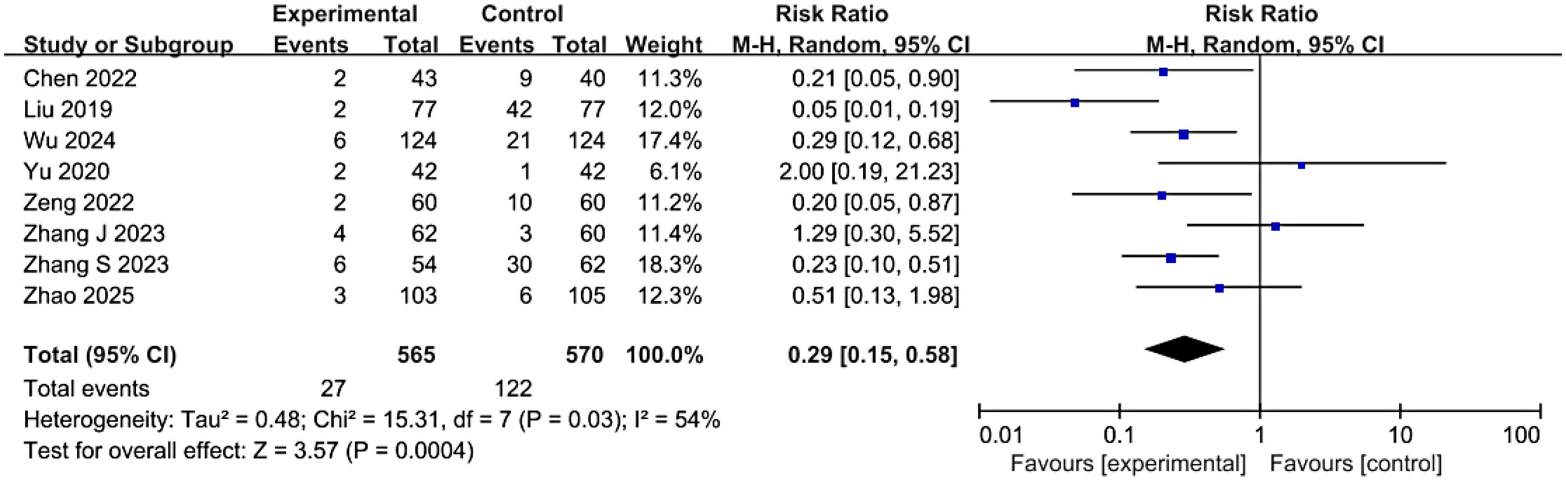
Forest plot for total postoperative complications.

All eight studies (14–21) reported postoperative complications, but definitions varied across studies. For the analysis of total complications, we included studies that reported the overall number of patients experiencing at least one complication. The pooled analysis using a random-effects model (due to moderate heterogeneity) showed that ERAS protocols significantly reduced the risk of total postoperative complications, with a risk ratio of 0.29 (95% CI: 0.15 to 0.58, *P* = 0.03; Figure 6). Moderate heterogeneity was present (I^2^ = 54%, *P* = 0.0004). Sensitivity analysis excluding the study with some concerns in risk of bias did not materially change the result (RR: 0.31, 95% CI: 0.15 to 0.67; I^2^ = 61%).

### Publication bias

Due to the limited number of included studies (<10), formal assessment of publication bias using funnel plots and Egger's test was not performed for any outcome, as the power of these tests would be too low to distinguish chance from real asymmetry.

## Discussion

This systematic review and meta-analysis of eight randomized controlled trials comprising 1,135 pediatric patients undergoing laparoscopic appendectomy evaluated the efficacy and safety of enhanced recovery after surgery (ERAS) protocols compared with traditional perioperative care. Our findings demonstrate that ERAS implementation significantly improves clinical outcomes across multiple domains. Specifically, ERAS protocols reduced postoperative hospital stay by a mean of 2.04 days (95% CI: −2.74 to −1.34), accelerated gastrointestinal function recovery with a mean reduction of 7.51 h in time to first flatus (95% CI: −11.00 to −4.03), alleviated postoperative pain with a mean reduction of 1.70 on the VAS (0–10 scale) (95% CI: −2.72 to −0.68), and reduced the risk of total postoperative complications by 71% (RR: 0.29, 95% CI: 0.15 to 0.58). It should be noted, however, that several individual ERAS components (e.g., shortened fasting, multimodal analgesia) have gradually become routine practice in some specialized centers. Nevertheless, in the RCTs included in this meta-analysis, the control groups uniformly received traditional perioperative care (e.g., prolonged fasting, routine catheterization, and passive analgesia), which still represents the standard of care in many institutions, particularly in emergency settings. Thus, the comparative effectiveness demonstrated here remains clinically relevant.

The magnitude of effect observed in this meta-analysis is consistent with previous systematic reviews in pediatric surgical populations. A systematic review by Arena et al. (22) examining ERAS in pediatric gastrointestinal surgery reported a reduction in hospital stay of approximately 2–3 days, which aligns closely with our finding of 2.04 days. Similarly, a meta-analysis by Zhang et al. (23) focusing specifically on acute appendicitis in children demonstrated significant improvements in postoperative recovery metrics comparable to our results. The consistency of these findings across different populations and settings strengthens the evidence base for ERAS implementation in pediatric appendicitis.

However, our results also reveal substantial heterogeneity across studies (I^2^ ranging from 54% to 99%), which warrants careful interpretation. Several factors likely contribute to this heterogeneity. First, the clinical spectrum of appendicitis varied considerably across included studies, ranging from uncomplicated (simple) to complicated (gangrenous, perforated, abscess) cases. This variability is clinically relevant, as complicated appendicitis is biologically associated with more severe inflammatory responses and prolonged baseline recovery, which may inherently create greater room for improvement through intensive perioperative interventions (3, 21). Second, the specific ERAS components varied substantially across studies. While all studies incorporated core ERAS elements such as shortened fasting, multimodal analgesia, and early mobilization, the intensity and combination of these elements differed. Notably, Zhao et al. (24) incorporated Child Life Specialist interventions—a psychological support approach that aligns with ERAS principles but may represent a distinct mechanism of action. The inclusion of such varied protocols may contribute to the observed heterogeneity while simultaneously reflecting the real-world flexibility of ERAS implementation. Third, differences in outcome measurement timing and definitions may influence heterogeneity. For pain scores, assessment time points ranged from 6 h to 24 h postoperatively, and some studies reported single time points while others reported multiple. Fourth, the absolute postoperative hospital stay in the ERAS groups across studies ranged from 4 to 9 days, which is longer than the 1–2 day discharge target often achievable for uncomplicated appendicitis in optimized day-surgery pathways. This likely reflects the inclusion of complicated cases (gangrenous, perforated, abscess) in several studies, as well as institutional variations in discharge criteria influenced by healthcare system factors (e.g., insurance coverage, parental confidence, and follow-up arrangements) rather than purely medical readiness. Our finding of a 2-day reduction represents a meaningful relative improvement, but achieving shorter absolute stays would require dedicated ambulatory protocols specifically designed for uncomplicated appendicitis, which was beyond the scope of this meta-analysis.

The findings of this meta-analysis have several important clinical implications. First, the consistent reduction in postoperative hospital stay by approximately 2 days suggests that ERAS protocols can substantially improve resource utilization and reduce healthcare costs. Given the high incidence of pediatric appendicitis, these benefits may translate into significant population-level cost savings (11). Second, the reduction in postoperative pain achieved through non-opioid multimodal analgesia aligns with current efforts to minimize opioid exposure in pediatric populations, thereby reducing the risk of opioid-related adverse events and dependency (25). Third, the accelerated gastrointestinal recovery and reduced complication rates support the safety and feasibility of early feeding and mobilization protocols, challenging traditional practices that delayed these activities until after flatus (1).

However, successful implementation of ERAS requires a multidisciplinary approach involving surgeons, anesthesiologists, nurses, and child life specialists, as well as engagement of patients and families (26). The variability in ERAS components across studies suggests that protocols may need to be adapted to local resources and patient characteristics while preserving core principles.

Several limitations of this meta-analysis should be acknowledged. First, the substantial heterogeneity observed across outcomes limits the precision of pooled estimates and suggests that the magnitude of benefit may vary depending on patient and protocol characteristics. Second, all included studies originated from China, which may limit the generalizability of findings to other healthcare settings with different practice patterns and resources. Third, the quality of evidence was limited by one study having “some concerns” in the RoB 2.0 assessment, primarily due to insufficient detail on intervention fidelity and lack of blinding for subjective outcomes. Fourth, the relatively small number of studies (*n* = 8) precluded formal publication bias assessment, leaving the possibility of unpublished negative results. Fifth, outcome definitions varied across studies, particularly for complications, which may have affected the consistency of pooled estimates. Sixth, none of the included studies reported long-term follow-up outcomes, such as chronic pain, quality of life, or readmission rates beyond the immediate postoperative period. Seventh, we did not perform subgroup analyses comparing uncomplicated vs. complicated appendicitis. Although disease severity is a key clinical determinant of recovery, the included studies varied substantially in their definitions and reporting of appendicitis type; some did not stratify by severity, while others exclusively enrolled complicated cases. Given this inconsistency, any pooled subgroup estimates would be unreliable and potentially misleading. Therefore, our results reflect the overall population of children with acute appendicitis, and the differential effects of ERAS by disease severity remain to be clarified in future studies with standardized severity classification.

Future research should address several gaps identified in this meta-analysis. First, large-scale, multicenter randomized controlled trials are needed to evaluate ERAS protocols across diverse geographic and healthcare settings to enhance generalizability. Second, future studies should adopt standardized ERAS protocols with clearly defined core elements to facilitate comparability and reduce heterogeneity. Third, investigation of long-term outcomes, including quality of life, chronic pain, and late complications, would provide a more comprehensive assessment of ERAS benefits. Fourth, cost-effectiveness analyses are warranted to quantify the economic impact of ERAS implementation and inform resource allocation decisions. Fifth, studies examining the optimal components of ERAS protocols—for example, comparing different multimodal analgesia strategies or early feeding protocols—would help refine recommendations for specific patient subgroups. Finally, given the publication of the first ERAS Society neonatal perioperative care guidelines in 2024 (7), future research should assess the applicability of these principles to the broader pediatric population and evaluate adherence to these evolving guidelines.

## Conclusions

This meta-analysis provides robust evidence that enhanced recovery after surgery protocols significantly improve clinical outcomes in children undergoing laparoscopic appendectomy. ERAS implementation reduces postoperative hospital stay, accelerates gastrointestinal function recovery, alleviates postoperative pain, and decreases complication risk compared with traditional perioperative care. While heterogeneity across studies warrants careful interpretation, the consistent direction of effects and the magnitude of benefits support the broader adoption of ERAS protocols in pediatric appendectomy. Future research should focus on standardizing protocol components, evaluating long-term outcomes, and assessing cost-effectiveness to further strengthen the evidence base and facilitate implementation across diverse clinical settings.

## Data Availability

The original contributions presented in the study are included in the article/Supplementary Material, further inquiries can be directed to the corresponding author/s.
